# Role of 3D Echocardiography in Cardiac Surgery: Strengths and Limitations

**DOI:** 10.1007/s40140-017-0226-5

**Published:** 2017-07-21

**Authors:** Edwin Wilberforce Turton, Jörg Ender

**Affiliations:** Department of Anesthesiology and Intensive Care Medicine, Heart Center Leipzig, Struempellstr 39, 04289 Leipzig, Germany

**Keywords:** Three-dimensional echocardiography (3DE), 3D transesophageal echocardiography (TEE), 3D transthoracic echocardiography (TTE), Intraoperative echocardiography, 3D data sets, 3D modes, Full volume (FV), Color flow Doppler (CFD), Real time (RT), Live, Zoom, Simultaneous multiplane/biplane/X-plane imaging, Cardiac surgery, Interventions, Transcatheter procedures, Echocardiographer, Spatial resolution, Temporal resolution, Guidelines and recommendations, Valvular surgery, Future developments, Strengths, Limitations

## Abstract

**Purpose of Review:**

This review aims to highlight the general and specific strengths and limitations of intraoperative 3D echocardiography. This article explains the value of real-time three-dimensional transesophageal echocardiography (RT 3D TEE) during cardiac surgery and cardiac interventions.

**Recent Findings:**

Recently published recommendations and guidelines include the use of RT 3D TEE. RT 3 D TEE provides additional value particularly for guidance during cardiac interventions (i.e., transcatheter mitral valve repair, left atrial appendix and atrial septal defect closures), assessment of the mitral valve in surgical repair, measurement of left ventricular outflow tract area for transcatheter valvular replacements, and estimating right and left ventricular volumes and function. The exact localization of paravalvular leakage is another strength of RT 3D TEE. The major limitation is the reduced temporal resolution compared to 2D TEE.

**Summary:**

Three-dimensional echocardiography is a powerful tool that improves communication and accurate measurements of cardiac structures.

## Introduction

Since the first use of transesophageal echocardiography (TEE) during cardiac surgery in 1979 [1], two-dimensional (2D) TEE has become a class I indication for perioperative use in almost every major cardiac surgical procedure [2].

Three-dimensional transesophageal echocardiography (3D TEE) was first available as an offline model after acquisition of multiple 2D examinations [3] that were very time consuming and user unfriendly [4]. Real-time 3D transesophageal echocardiography (RT 3D TEE) using a matrix probe evolved during the late 2000s [5] and led to the widespread use of this technique in cardiac surgery today.

This article highlights the strengths and limitations of RT 3D TEE in daily clinical practice during cardiac surgery and interventional cardiac procedures. This article is divided into two parts. The first discusses the strengths and the limitations of 3D echocardiography in comparison to 2D echocardiography (2DE), and the second outlines the specifics of RT 3D TEE looking at different cardiac structures.

## Cardiac Surgery and Three-Dimensional Echocardiography

### General Strengths and Limitations

Two-dimensional echocardiography to visualize the 3D structure of the heart requires cognitive reconstruction from multiple 2D images. This is no longer required in 3D echocardiography. Further, while 2D echocardiography only reveals cross-sectional views of the heart, 3D echocardiography is more akin to a Cartesian coordinate system [6••], enabling us to display the anatomic view of the heart. This simplifies communication between physicians and allows for better orientation, which is particularly helpful for image-guided interventional procedures [2].

Because ultrasound propagation is equal in both 2D and 3D echocardiography, the use of more Cartesian coordinate-like data to improve spatial orientation leads to reduced temporal resolution. Practically, the echocardiographer using 3D echocardiography has to decide between the optimal temporal and spatial resolutions when imaging specific cardiac structures.

Both temporal and spatial resolutions play a role in the quality of images produced by 3D echocardiography. Images need to be of adequate quality to allow qualitative and quantitative assessment. Temporal resolution is displayed as volumes per time unit (volume rate), and this can be increased by decreasing the volume size (sector) imaged. Spatial resolution can be improved by reducing the volume acquired or by increasing the number of scan lines through the volume. Increasing the number of scan lines for a specific volume will reduce its temporal resolution.

Different 3D modalities are available (i.e., wide-sector zoom, narrow sector, full volume (FV) mode with and without color Doppler). The technical aspect of these different modalities will not be discussed in detail in this article, but it is explained elsewhere [7••].

The inherent physical properties of ultrasound and with that the limitations of ultrasound imaging in general are equally applicable to 3D and 2D echocardiography. Additionally, there are specific 3D-based artifacts such as stitching artifacts, dropout, “blurring” or thickening, blooming, and rail road artifacts [8–10]. Dropout artifacts can mimic valvular perforations and periprosthetic leaks [11].

Another limitation comes with displaying 3D echocardiographic images on a 2D screen. In doing so, colors are used to give a sense of depth and reality [12••]. Therefore, accurate measurements cannot be made in RT 3D mode [13].

### Specific Strengths and Limitations (See Table 1)

#### Left Ventricle Volume and Function

Simultaneous multiplane mode allows the recommended biplane measurement for ejection fraction of the same heartbeat, whereas in 2D TEE assessment, it can only be performed in two different heartbeats [14].

**Table 1:** Structure-specific 3D mode and clinical utility

Structure	View	3D Mode	Clinical utility [7]
Mitral valve	ME 0–120° ± CFD	Multiplane, Zoom, FV	Morphology (pathology), function, quantification—EROA and MVA
Aortic valve	ME SAX and LAX views ± CFD	Multiplane, Zoom, Live	Valvular surgery and interventions esp. transcatheter valvular replacements (TAVR)
Tricuspid valve	ME 4C, 0°–30°, TG SAX (40°)	Multiplane, Zoom, Live	Unstudied (transcatheter procedures experimental)
Pulmonary valve	UE 90°, ME 3C 120° ± CFD	Multiplane, Zoom	Limited visualization with TEE
IAS	ME 0° view	Multiplane, Zoom, FV	Atrial septal defect, patent foramen ovale
LV	ME 0°–120° views	Multiplane, FV	Volume, mass, EF, dysynchrony, deformation parameters
RV	ME 0°–120°	Multiplane, FV	Volume, EF
LAA	ME 0°, 45°, 60°, 90°	Multiplane, Live, Zoom	Thrombus

Modified from [14]

*CFD* color flow Doppler, *EROA* effective orifice area, *EF* ejection fraction, *IAS* interatrial septum, *4C* four chamber, *FV* full volume, *LAX* long axis, *ME* midesophageal, *MVA* mitral valve area, *SAX* short axis, *TEE* transesophageal echocardiography, *TG* transgastric

With FV mode and multiple beat acquisitions, the left ventricular volume and function can be more accurately determined by 3D than by 2D echocardiography when compared to other image modalities like multidetector computed tomography (MDCT) or cardiac magnetic resonance imaging (CMRI) [15••]. Three-dimensional echocardiography is more accurate than both contrast enhanced 2D echocardiography and 2D echocardiography alone in the measurement of LV ejection fraction, but still underestimates LV volumes compared to CMRI [16]. The strength of left ventricular assessment with 3D echocardiography is that no geometric assumption is necessary and that it is unaffected by foreshortening [14]. The limitations are the lower temporal resolution and that no normal values for 3D TEE are available so far.

Image acquisition time to assess LV function and volume is equally between both 3D and 2D. But, analysis of the 3D data set takes on average 2 min longer [17]. In addition, a special software package for 3D analysis, not automatically included when buying a 3D system, is required.

Especially advantageous in the perioperative setting, nearly all left ventricular dimensions can be measured based on a single FV data set when using 3D TEE. This may be important in cardiac surgery when the patient is either unstable or there is not enough time for a comprehensive 2D TEE examination [18]. Multislice imaging of the ventricle can be performed, and regional wall motion and function can be assessed using this single data set.

#### Right Ventricle Volume and Function

The complex geometry of the right ventricle makes it impossible to evaluate right ventricular ejection fraction (EF) using 2D TEE. For experienced echocardiographers with access to special software, it is recommended to use 3D-derived right ventricular EF [14]. Good accuracy and correlation for 3D right ventricular function in cardiac surgical patients with preoperative 3D transthoracic echocardiography examinations have been shown in the perioperative setting [19, 20]. The main limitations are the need for special software and image quality.

#### Mitral Valve

The non-planar anatomy of the mitral valve (MV) complex lends itself to 3D assessment. It is more intuitive to assess the MV using 3D echocardiography than to reconstruct this three-dimensional structure using multiple 2D views [21, 22]. The MV can be displayed from the left atrial side and the left ventricle side using one 3D echocardiography image or data set [7••]. Assessment of the mechanism of MV pathology can be done accurately [23, 24]; and 3D TEE is especially superior to 2D TEE in viewing complex MV anatomy and determining pathology [25••, 26].

Three-dimensional MV assessment especially for MV repair surgery can be used to predict the probability of successful MV repair and to assess the result after repair [6••, 27], both leading to optimal outcomes after surgery [28].

#### Quantification of Mitral Valve Disease ( Mitral Regurgitation and Mitral Stenosis)

Color flow Doppler in FV mode can be used to measure the VC area (VCA) and the proximal isovelocity surface area (PISA) by planimetry [24••, 29], overcoming the caveats of flow-derived calculation in 2D TEE [30]. The most important limitations are the temporal and spatial resolutions and cumbersome offline analysis [24••].

Three-dimensional TEE is remarkable in its ability to assess commissural fusion [31] in rheumatic MV disease, and its measurement of the MV area is as accurate as with 2D TEE [32].

#### Localization of Paravalvular Leakage

Paravalvular leaks can be exactly located [33] and quantified [34].

#### Aortic Valve

Three-dimensional echocardiography can accurately display the anatomy of the aortic valve in most patients and simplify communication between physicians [35], but in some patients, the imaging is limited due to acoustic shadowing [8] and echo dropout especially in degenerative calcified aortic cusps [9••].

Subvalvular aortic membranous structures as well as dynamic left ventricular outflow tract (LVOT) obstruction can be diagnosed by 3D echocardiography [36], but low temporal resolution may miss the dynamic obstruction [37].

Assessment of left ventricular outflow area with 3D echocardiography has been shown to correlate better with multislice computed tomography (MSCT) compared to 2D echocardiography [38, 39] and is, therefore, recommended [40••]. Sizing of transcatheter (transcatheter valvular replacements (TAVR)) valves is discussed in the section on transcatheter procedures/interventional procedures.

Pathology can be assessed by 2D and additionally by 3D TEE before aortic root, valve-sparing aortic, or valve repair surgery [24••, 41].

#### Quantification of Aortic Valve Disease (Aortic Regurgitation and Aortic Stenosis)

In aortic stenosis, 3D TEE can be used to measure left ventricular outflow area and diameter [40••]. Semi-automated 3D measurements seem to be better as compared to direct planimetry [42]. The underestimation of the LVOT area in the 2D method may lead to overestimation of aortic stenosis. Therefore, 3D echocardiography may have an impact on surgical decision making in these cases [39, 43]. Recent recommendations suggest the use of 3D TEE or MSCT to measure the LVOT and to use this measurement in the quantification of aortic valve area [40••].

The use of 3D color flow is not recommended in quantification of aortic regurgitation due to the low temporal and spatial resolutions, except in patients with multiple jets [41]. In these patients, overestimations or underestimations of the jet size may occur.

#### Tricuspid Valve

The assessment of the tricuspid valve (TV) and its pathology has to include assessment of the right ventricle and right atrium. Three-dimensional echocardiography allows accurate evaluation of right ventricle function and volumetric assessment with specialized and costly software.

Using biplane 3D TEE mode at different levels of the esophagus and stomach makes it possible to accurately identify the different leaflets [44•].

Color flow Doppler (CFD) can be added to the FV set to more accurately assess insufficiency or stenosis of the valve [23, 44]. Vena contracta area (VCA), especially in the presence of multiple jets, can be assessed by 3D color Doppler echocardiography [24••]. Nevertheless, over- or underestimation may occur with inadequate spatial resolution. The three-dimensional proximal isovelocity surface area (PISA) method in functional tricuspid regurgitation is clinically feasible and more correct than the 2D PISA method [45]. Planimetry of the tricuspid annulus can be done accurately and can detect eccentric dilatation in the presence of severe tricuspid regurgitation [46].

The greater distance between the probe and the TV and the non-perpendicular alignment make imaging of the TV by RT 3D TEE difficult [24••].

#### Pulmonary Valve

The thin leaflets of the pulmonary valve (PV) are situated in the far field from the TEE probe, and an echo dropout artifact is frequently encountered. Therefore, RT 3D TEE has no advantage over 2D TEE in the clinical evaluation of the PV.

#### Atria

Three-dimensional echocardiography is more accurate in measuring left atrial (LA) and right atrial (RA) volumes and compares well with CMRI measurements [14]. Using RT 3D TEE, the atria cannot be fully measured due to the close proximity of the TEE probe to the atria. However, nearby structures can be visualized anatomically in detail (i.e., pulmonary vein ostia into the LA, vena cavae ostia, coronary sinus ostium, crista terminalis, eustachian valve, and fossa ovalis in the RA) [47].

#### Great Vessels

Three-dimensional TEE is more sensitive and detects cases of aortic aneurysms and dissections that are missed with 2D TEE. The dissection flap appears in 3D TEE as a tissue flap. The linear image of the dissection membrane can be mistaken for an artifact in a 2D examination. The involvement of the right coronary artery ostium in aortic dissections is more accurately diagnosed by 3D TEE [48].

#### Interventional Cardiac Procedure

The complexity of cardiac surgery and interventional procedures is increasing, and intraoperative and intraprocedural three-dimensional imaging is necessary to guide these [49, 50].

#### Transcatheter Valvular Replacements

While MSCT is the gold standard for sizing the aortic valve, 3D TEE is an accurate alternative in units where MSCT is not available or in situations where it is contraindicated [50–52]. The simultaneous multiplane mode can be used to correctly align the long axis of the aortic valve to measure the annulus accurately [52•]. Both simultaneous multiplane mode and RT 3D TEE are used during these procedures. With 3D TEE, annular distances to both coronary arteries can be measured using the multiplanar reconstruction (MPR) mode [50, 52, 53]. Assessing the position of the valve prosthesis on the balloon is better done with 3D TEE, because it can guide the correct positioning of the prosthesis in relation to the valve annulus and surrounding structures. The path of the guide wire through the LV and around the subvalvular apparatus of the MV is better visualized with RT 3D TEE [54]. Three-dimensional TEE can also be used to assess post-implantation results, including the occurrence of intravalvular and paravalvular regurgitation [50, 52].

#### Paravalvular Leakage Diagnosis

Live 3D zoom TEE images can be used to correctly localize paravalvular leaks. Care should be taken not to mistake echo dropout artifacts as paravalvular leaks. The size of the dehiscence can be determined by acquiring a FV set with CFD to confirm leakage and to measure its size [50].

#### Transcatheter Mitral Valve Repair

Guidance RT 3D TEE and simultaneous multiplane mode are strongly recommended for guidance of transcatheter edge to edge MV repair with the MitraClip® [55–58].

The Cardioband® direct annuloplasty MV repair is done with fluoroscopic and 2D/3D TEE guidance. Simultaneous biplane and 3D TEE are used for transseptal puncture and RT 3D TEE (wide-sector zoom) to assess anchoring position and final adjustment [59].

RT 3D TEE can be used in addition to 2D TEE and fluoroscopy to help with coronary sinus identification during the indirect coronary sinus annuloplasty technique [60].

Mitral stenosis can be treated by balloon valvotomy if found suitable for the procedure. Three-dimensional TEE can be used to assess the mitral commissural fusion to further determine suitability for this procedure. Three-dimensional TEE with its simultaneous biplane mode is used for correct transseptal puncture [57, 61].

#### Left Atrial Interventions

Intra-atrial Septum and Left Atrial Appendix Three-dimensional TEE is superior to 2D TEE in the spatial information that it provides to delineate the variant anatomy of a patent foramen ovale (PFO) and of atrial septal defects (ASD). The en-face image of the intra-atrial septum allows for spatial orientation and counting of defects. RT 3D TEE is also very suitable to guide the intervention. Offline MPR is useful for correct measurement of ASD size through precise alignment, can indicate rim deficiencies, and allows measurement relationships to important structures like the aorta [62•].

The sizing of the left atrial appendix (LAA) can be done by RT 3D TEE [63]. Two-dimensional TEE significantly underestimates the maximal LAA orifice compared to RT 3D TEE preclosure and during the procedure [64]. Additionally, RT 3D TEE is useful in ruling out a LAA thrombus [50].

#### Infective Endocarditis and Cardiac/Aortic Sources of Embolism

Infective endocarditis (IE) and its complications can readily be diagnosed and further clarified by the use of intraoperative 2D TEE. Three-dimensional TEE FV and real-time modes reveal pathology that is not readily seen with 2D echocardiography [65]. There is a good correlation between 3D TEE assessment of pathology and corresponding operative findings [66]. Morphology and size of valvular vegetations can be assessed, superior to 2D TEE [67].

Three-dimensional and multiplane imaging are suggested to confirm or exclude cardiac masses in the cardiac chambers [68].

Volumetric assessment of cardiac masses can be performed with 3D echocardiography, and further detailed assessment is possible: looking at attachment and detailed morphology [69].

Thromboembolism from the thoracic aorta and atheromatous plaque in the aortic arch should be visualized in 3D and multiplane images [70].

### Future Developments

More complex percutaneous and transapical valve procedures are attempted and periprocedural 3D TEE guidance may also be useful in upcoming interventions [71–73].

Automated or semi-automated software for quantification of the MV, for example, is feasible [74, 75], but the benefit of this sophisticated software needs further investigation before routine clinical use.

Hybrid imaging techniques with the different imaging modalities merged and used at the same time will also improve the capabilities of 3D imaging [76]. Combined TEE and fluoroscopy merged/fused images may play a role in improved outcomes [28, 54].

Three-dimensional strain and strain rate as parameters of myocardial deformation for the assessment of the left ventricle, right ventricle, and left atrium will play an important role in chamber quantification in the future [77, 78•].

## Conclusion

For the first time, RT 3D TEE allows for the anatomical display of most cardiac structures and pathologies, therefore facilitating improved communication between physicians. It additionally allows for more accurate quantification of some stenotic as well as regurgitant pathologies. Further development in 3D technology and the definition of normal values are the next steps to increase the clinical value of RT 3D TEE.
